# Genetic diversity of *Gymnocypris chilianensis* (Cypriniformes, Cyprinidae) unveiled by the mitochondrial DNA D-loop region

**DOI:** 10.1080/23802359.2021.1906172

**Published:** 2021-04-01

**Authors:** Yong-Jie Wang, Jun-Hao Lu, Zhe Liu, Jiu-Pan Zhang

**Affiliations:** College of Animal Science and Technology, Gansu Agricultural University, Lanzhou, Gansu, China

**Keywords:** *Gymnocypris chilianensis*, *D-loop*, genetic diversity, genetic differentiation

## Abstract

In order to analyze the genetic diversity and genetic differentiation of *Gymnocypris chilianensis*, *D-loop* region of the mitochondrial DNA was sequenced in 50 individuals of *G. chilianensis* obtained from 2 geographic locations (Heihe River and Shule River) and 25 individuals of *G. przewalskii* (Qinghai Lake). Twenty-five homologous sequences of another *G. eckloni* (Yellow River) downloaded from GenBank were analyzed together. The sequences were analyzed by using the MEGA (version 7.0) and DnaSP (version 6.0) software. The results revealed that 82 haplotypes were detected among 100 individuals. The haplotype diversity (*Hd*) and nucleotide diversity (*Pi*) of *G. chilianensis* of the Shule River were 0.963 ± 0.029 and 0.00414 ± 0.00069, which were lower than those of 3 other populations. The genetic distance of *G. chilianensis* in both Heihe River and Shule River was 0.0013. The genetic distances between the 2 *G. chilianensis* populations and the *G. eckloni* were 0.0148 and 0.0141, respectively. Population differentiation values (*Fs*t) and gene flow (*Nm*) showed that 4 population had occurred obvious genetic differentiation (*Fst*: 0.20811 ∼ 0.98863. *p* < 0.01; *Nm* < 1). Compared with *G. przewalskii* and *G. eckloni*, the differentiation degree was more significant between Heihe River *G. chilianensis* and Shule River *G. chilianensis* (Fst = 0.98863, *p* < 0.01; Nm = 0.00287). Maximum Likelihood (ML) phylogenetic tree showed that *G. chilianensis* had further genetic distance with *G. eckloni* and *G. przewalskii*. In conclution, *G. chilianensis* (HH&SL) had lower genetic diversity and further genetic distance than *G. przewalskii* (QH) and *G. eckloni* (YL). We suggest strengthen the protection of genetic resources of *G. chilianensis*.

## Introduction

*Gymnocypris chilianensis* is a species of the genus *Gymnocypris* which belongs to the subfamily Schizothoracinae of the family Cyprinidae. It is mainly distributed in the Shiyang River, Heihe River and Shule Rivers, inland river basin of Hexi Corridor in Gansu province of China (Li and Chang 1974; Wu and Wu 1992). The Hexi Corridor is located at the border of the Tibetan Plateau, the Loess Plateau and the Inner Mongolian Plateau. The three inland rivers in the Hexi Corridor originate from the Qilian Mountains. Water resources for supply of three rivers mainly depend on snow and rain water from the Qilian Mountains (Zhang et al. 2015). *Gymnocypris chilianensis* has delicious meat, rich nutritional value, and high economic value. In recent years, the distribution area and the wild resources of *G. chilianensis* are decreasing dramatically, which is mainly influenced by natural and artificial factors. As a result, its genetic diversity is diminishing progressively. The adaptability of species is positively correlated with genetic diversity. Lack of genetic diversity poses a huge threat to species living in anthropogenically disturbed habitats (Xiao et al. 2013). To protect and utilize the resources of *G. chilianensis*, it is necessary to investigate the genetic diversity and population structure of *G. chilianensis.*

At present, there are few studies on the genetic diversity and population structure of *G. chilianensis*. Zhao et al. (2011) used mitochondrial DNA sequence data (a control region and the cytochrome *b* gene; 1894 bp) to assess the phylogeographic structure of *G. chilianensis* in the inland river watersheds of the Hexi Corridor in Gansu Province, China. Moreover, we have studied the genetic diversity and taxonomic status of *G. chilianensis* based on the mitochondrial DNA cytochrome *b* gene (Zhang et al. 2015). The results showed that both the haplotype diversity and nucleotide diversity were lower than the other populations. Analysis of sequence differences indicates that G. chilianensis is sufficiently diverged from *G. przewalskii* and *G. eckloni* to the extent that it has reached species level, it provides a reference basis for G. chilianensis to be regarded as an independent species of *Gymnocypris*. As a non-coding fragment of mtDNA, the D-loop region is not subject to the pressure of coding selection, and has long been considered to have the fastest mutation rate in the mitochondrial genome. Compared with cytochrome *b* gene of mtDNA, it is more suitable for the genetic diversity, population genetic structure and phylogeographic analysis (Cann et al. 1984). Dai et al. (2010) have studied the genetic diversity of Wujiang River population of *Schizothorax kozlovi* by using mtDNA *D-loop* region. Its genetic diversity was found to very low and the protection for the population is necessary. Zhang et al. (2013) used the *D-loop* region to study the four populations of *Gymnocypris przewalskii* (Lake Qinghai, Lake Keluke, Ganzi River and Cao Dalian). It was concluded that *Gymnocypris przewalskii* has high genetic diversity, and there is a certain degree of genetic differentiation among populations, especially the Keruke Lake population has been highly differentiated, but the level of genetic diversity is very low, it should be priority to protect.

In this study, we analyzed the genetic diversity and population structure by focusing on *G. chilianensis* and using the mtDNA D-loop gene as a marker, with the aim of providing a theoretical basis for resource conservation and utilization of *G. chilianensis.*

## Materials and methods

### Study area

A total of 50 individuals of *G. chilianensis* were collected, among which 25 individuals each were obtained from Heihe (HH)(N100°30′; E38°57′) and Shule (SL) (N96°13′; E42°21′) rivers. Besides, 25 individuals of *G. przewalskii* were collected from Qinghai Lake (QH) (N99°38′; E36°32′).

### Sample collection

All individuals were identified based on morphological characteristics (Li and Chang 1974; Zhao 2005). A part of caudal fin was collected from each individual, preserved in 95% ethanol and stored at −20 °C in the Fisheries Laboratory of Gansu Agricultural University. Genomic DNA was extracted by proteinase K digestion followed by a standard phenol-chloroform method (Green and Sambrook 2012). 25 *D-loop* region sequences of *G. eckloni* (YL) which were derived from the mainstream of the Yellow River in Qinghai Province of China, were downloaded from the GenBank database (GenBank accession No. FJ601074-FJ601098)

### Amplification, cloning and sequencing

*D-loop* sequences were amplified with the new specific primers: F5′-GGGATATGTCATCCTTTATGG-3′, R5′-GGGTTTGACAAGAATAACAGG-3′. The PCR reaction was performed in a total volume of 50 μL containing 50 ng of template DNA, 100 µM of dNTPs (Takara, Dalian, China), 2.5 mM of 1 × PCR reaction buffer, 1 U of Taq DNA polymerase (Takara), 0.4 µM of each primer and 19.3 μL of ddH_2_O. PCR was carried out under the following conditions: an initial denaturation at 95 °C for 3 min, followed by 30 cycles of denaturation at 94 °C for 30 s, annealing at 57 °C for 30 s, and extension at 72 °C for 90 s, followed by a final extension at 72 °C for 10 min.

Subsequently, PCR products were analyzed using 1% agarose gel and purified using the Universal DNA Purification Kit (TianGen, Beijing, China). Purified PCR products were subsequently cloned into pMD-19T Simple Vector (TaKaRa) and the ligation products transformed into DH5α Chemically Competent Cell *Escherichia coli* (TianGen) according to the manufacturer’s instructions. Colonies of *E. coli* harboring recombinant clones were screened for the presence of inserts of the expected size by LacZ blue-white selection and identified by double digestion with *Eco*RI and *Pst*I. The positive recombinant clones were sequenced from both directions (Sangon Biotech, Shanghai, China).

### Data analysis

The *D-loop* sequences were separately aligned and trimmed to equal lengths using the BioEdit program (http://www.mbio.ncsu.edu/bioedit/bioedit.html) and ClustalX 2.1 program (http://www.clustal.org/). The nucleotide composition and genetic distance among four populations (the average genetic distances based on Kimura’s two-parameter model) were computed using MEGA 7.0 software (http://www.megasoftware.net/), with the standard errors estimated by bootstrapping using 1000 replicates (Kimura 1980). Genetic diversity parameters were estimated using DnaSP 6 software (http://www.ub.edu/dnasp/). For phylogenetic analysis, *Gymnodiptychus pachycheilus* (GenBank accession No. of *D-loop*: FJ601172) and *Ptychobarbus dipogon* (GenBank accession No. of *D-loop*: FJ601178) were taken as the out-group, Maximum Likelihood (ML) phylogenetic tree based on *D-loop* haplotypes was constructed using MEGA 7.0, and statistical support was estimated using 1000 bootstrap replicates. Haplotype network (median-joining) was generated using Network 5 software (http://www.fluxus-engineering.com/) (Liao et al. 2016; Gao et al. 2017; Nisar et al. 2019).

## Results

### Base composition

A total of 100 homologous sequences of 747 bp were used for the base composition analysis. The average base composition was T = 32.1%, C = 21.9%, A = 31.9%, and G = 15.1%. The base compositions of *G. chilianensis*, *G. eckloni* and *G. przewalskii* were very similar in the *D-loop* region, indicating that the base composition did not change with the variation. Average Homoplasy of 4 population compared with each other was 98.1%.

### Gene mutation

A total of 100 homologous sequences of 747 bp were used for the genetic analysis of gene mutations, and a total of 124 mutation sites were detected, accounting for 16.4% of the total analyzed sites, including 119 transitions and 9 transversions. The transition/transversion ratio was 13.2 and significantly greater than 2.0. Therefore, the mutagenesis was not needed saturated and weighted analysis in the phylogenetic analysis (Knight and Mindell 1993).

### Genetic diversity

A total of 82 haplotypes were recovered from 100 aligned sequences and the haplotype diversity and nucleotide diversity indices were shown in Table 1. The haplotype diversity of the Qinghai Lake *G. przewalskii* population was the highest (*Hd* = 0.997), compared with the lowest haplotype diversity of the Yellow River *G. eckloni* (*Hd* = 0.943). The nucleotide diversity of the Qinghai Lake *G. przewalskii* was the highest (*Pi* = 0.00831), compared with the lowest nucleotide diversity of the Shule River *G. chilianensis* (*Pi* = 0.0014).

**Table 1.** Genetic diversity indices of the four *Gymnocypris* populations studied.

Population	Sample size	Hp	V (%)	S	Hd (SD)	Pi (SD)
HH	25	20	80	36	0.973 (0.022)	0.00626 (0.00072)
SL	25	20	80	35	0.963 (0.029)	0.00414 (0.00069)
QH	25	24	96	55	0.997 (0.012)	0.00831 (0.00132)
YL	25	18	72	22	0.943 (0.037)	0.00745 (0.00050)

Note: HH = Heihe River *G. chilianensis*; SL = Shule River *G. chilianensis*; YL = Yellow River *G. eckloni* and QH = Qinghai Lake *G. przewalskii*. S: number of polymorphic (segregating) sites; Hd: haplotype (gene) diversity; Pi: nucleotide diversity; SD: standard deviation.

### Genetic differentiation

Genetic distance within and among populations was estimated using the Kimura’s two-parameter model, with values ranging from 0.0042 to 0.0084 and from 0.0013 to 0.0148, respectively (Table 2), suggesting that the genetic distances among populations were higher than within populations. The genetic distance between the Heihe River *G. chilianensis* population and the *G. eckloni* population were found to be the highest (0.0148), followed by the genetic distance between Heihe River *G. chilianensis* population and the *G. przewalskii* population (0.0143), while the genetic distance between the Heihe River *G. chilianensis* population and the Shule River *G. chilianensis* population were the lowest (0.0013).

**Table 2.** Genetic distance among the four *Gymnocypris* populations (below diagonal) and genetic distance within populations (in bold, above diagonal).

Group	HH (SD)	SL (SD)	QH (SD)	YL (SD)
HH	0.0063 (0.0015)			
SL	**0.0013 (0.0012)**	0.0042 (0.0007)		
QH	**0.0143 (0.0060)**	**0.0137 (0.0059)**	0.0084 (0.0015)	
YL	**0.0148 (0.0062)**	**0.0141 (0.0061)**	**0.0013 (0.0007)**	0.0075 (0.0019)

Note: HH = Heihe River *G. chilianensis*; SL = Shule River *G. chilianensis*; YL = Yellow River *G. eckloni* and QH = Qinghai Lake *G. przewalskii*. SD: standard deviation.

DNASP 6 software was used to calculate the *Fst* and *Nm* among the 4 populations, and the results were shown in Table 3. *Fst* (*Fst* > 0.25) and *Nm* (*Nm* < 1) of the four populations reached the level of high differentiation, with the highest *Fst* and the lowest *Nm* between Heihe River *G. chilianensis* and Shule River *G. chilianensis*, the lowest *Fst* and the highest *Nm* between Qinghai Lake *G. przewalskii* and Yellow River *G. eckloni*.

**Table 3.** *Fst* (below diagonal) and *Nm* (above diagonal) among the 4 *Gymnocypris* populations.

Group	HH	SL	QH	YL
HH		0.00287	0.00369	0.00342
SL	0.98863 (*p* < 0.01)		0.07568	0.06762
QH	0.98545 (*p* < 0.01)	0.76761 (*p* < 0.01)		0.95128
YL	0.98648 (*p* < 0.01)	0.78710 (*p* < 0.01)	0.20811 (*p* < 0.01)	

Note: HH = Heihe River *G. chilianensis*; SL = Shule River *G. chilianensis*; YL = Yellow River *G. eckloni* and QH = Qinghai Lake *G. przewalskii*.

### Matrilineal genealogical analysis

**Figure 1.**
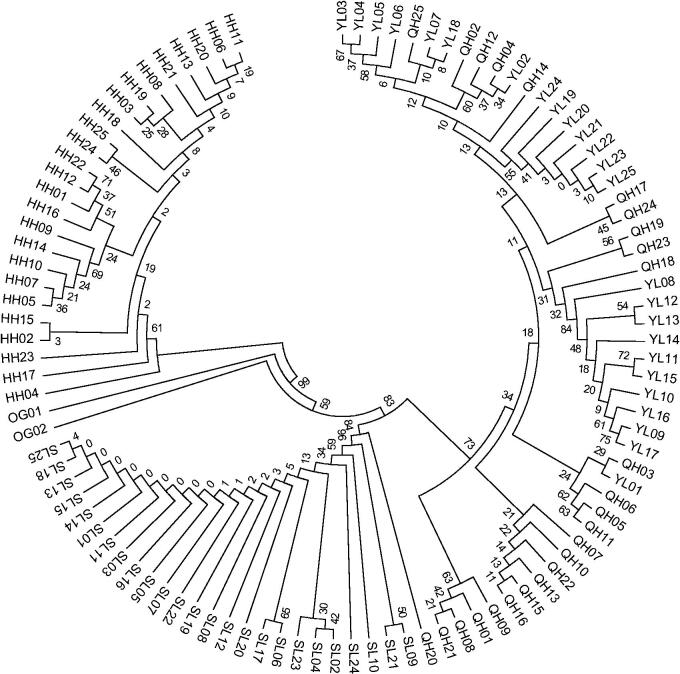
Maximum Likelihood (ML) phylogenetic tree of the 78 D-loop haplotypes from the Gymnocypris populations. Note: HH = Heihe River *G. chilianensis*, SL = Shule River *G. chilianensis*, YL = Yellow River *G. eckloni*, QH = Qinghai Lake *G. przewalskii*, OG01 = Yellow River *Gymnodiptychus pachycheilus*, OG02= Yellow River *Ptychobarbus dipogon.*

The matrilineal genealogical analysis showed that 82 haplotypes diverged into two main branches. One main branch contains the Heihe River *G. chilianensis*. Another branch contains Shule River *G. chilianensis*, *G. przewalskii* and *G. eckloni* which the Shule River *G. chilianensis* from a haplotype clade, bootstrap values were 84%, *G. przewalskii* and *G. eckloni* from another haplotype clade, bootstrap values were 73% (Figure 1).

### Haplotype network analysis

**Figure 2.**
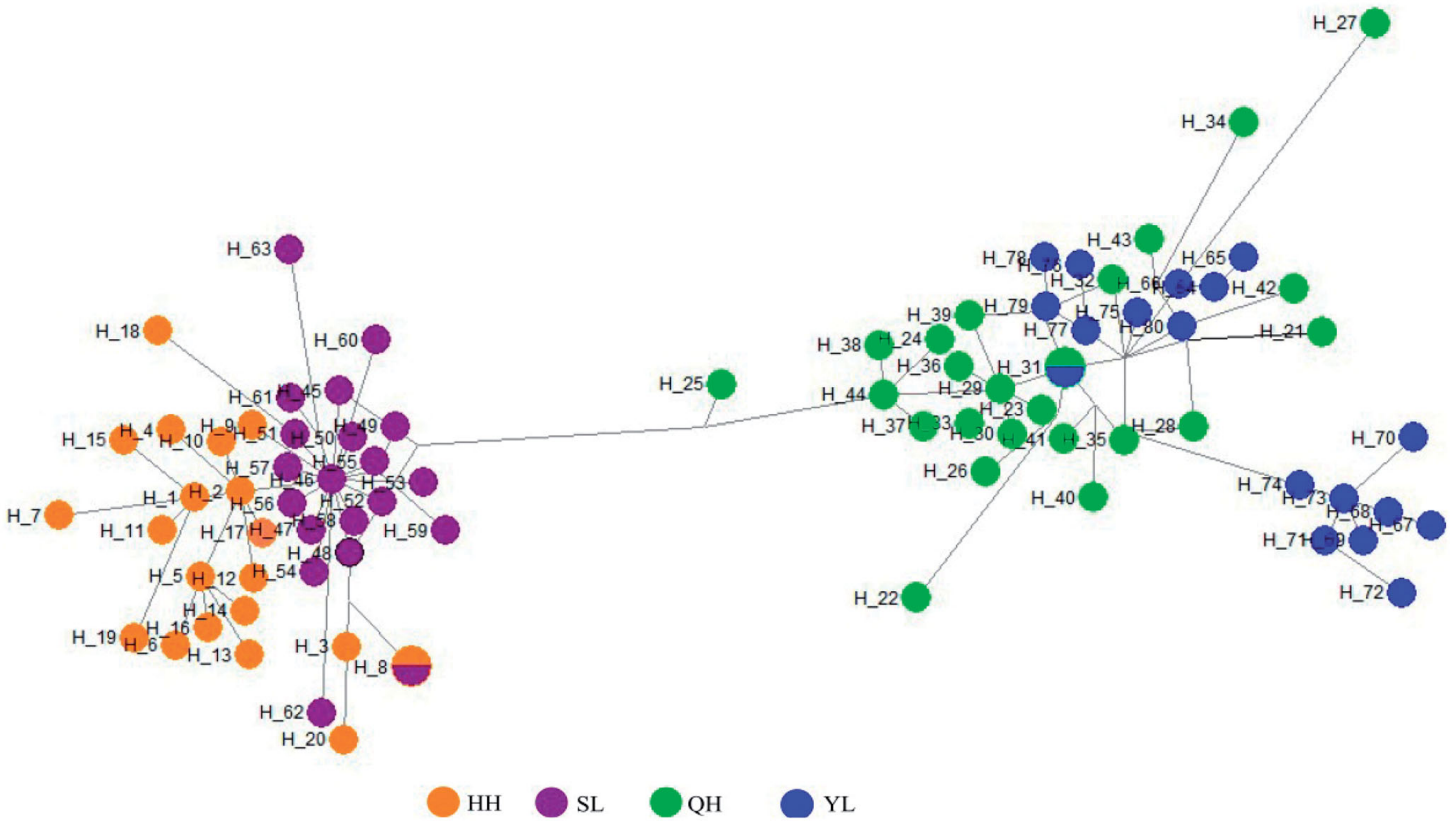
Median joining network of the haplotypes reconstructed in present study for Gymnocypris fish. Note: A circle represents a haplotype, the blue stand for *G. eckloni* (YL), the green stand *G. przewalskii* (QH), orange and purple stand for *G. chilianensis* (HH&SL). HH = Heihe River *G. chilianensis*, SL = Shule River *G. chilianensis*, YL = Yellow River *G. eckloni* and QH = Qinghai Lake *G. przewalskii*

To understand the relationships of haplotypes, the median joining network was constructed for the identified haplotypes. All the haplotypes were clustered into two big groups (haplogroups a and b). *G. chilianensis* (HH&SL) clustered on haplogroups a, *G. eckloni* (YL) and *G. przewalskii* (QH) clustered on haplogroups b and some haplotype cross distribution each other (Figure 2).

## Discussion

Haplotype diversity (Hd) and nucleotide diversity (Pi) are important indicators of population genetic variation. They are a valuable indicator for estimating the abundance or scarcity of the genetic diversity of a population (Li et al. 2011). In this study, the *D-loop* region sequence analysis showed that the haplotype diversity (Hd =0.9970) and nucleotide diversity (*Pi* = 0.00831) of *G. przewalskii* population were the highest among the four groups. The nucleotide diversity index of the Shule River *G. chilianensis* population was 0.00414, which was lower than the other populations analyzed in our study, but still well above the research results on the Shule River *G. chilianensis* (*Pi* = 0.0002) by Zhao et al. (2011), and much higher than the result of research on the *Spinibarbus yunnanensis* (*Pi* = 0.0014) by Li and Lü∨ (1998). According to the range of *Pi* of genetic diversity proposed by Lan and Shi (1993), the genetic diversity of the Shule River *G. chilianensis* population was lower (*Pi* < 0.0047). Moreover, the combination of high haplotype diversity (*Hd* = 0.9630) with low nucleotide diversity (*Pi* = 0.00414) of the Shule River *G. chilianensis* population reveals possible mechanisms for the formation of this population, which multiply rapidly from a small effective fish population (Avise 2000). Our result was similar to the observed in *Anabarilius grahami* by Yang et al. (2008).

The genetic distance based on the *D-loop* region indicates that the genetic distances among populations were higher than within populations. Due to the lack of clear genetic distance criteria, which based on *D-loop* region, we cannot measure whether it reached the species differentiation level between individuals. In present study, the genetic distance based on the *D-loop* region sequence can only show the relationship between *G. chilianensis* and *G. eckloni* is distant, and the relationship between *G. chilianensis* and *G. przewalskii* is distant. The present result is accorded with the viewpoint of Zhang et al. (2015), which means that the genetic relationship between *G. chilianensis* and *G. przewalskii* and *G. eckloni* is distant. *Fst* is an important indicator of genetic differentiation among populations. 0< *Fst* < 0.05 indicates that there is no differentiation among its subgroups. 0.05< *Fst* < 0.15 indicates that there is moderate differentiation; 0.15< *Fst* < 0.25 indicates that there is high differentiation. In this study, we found that the *Fst* of the four populations reached 0.20811 ∼ 0.98863 (*p* < 0.01), which indicated that obvious genetic differentiation had occurred in the four populations. *Nm* < 1 between populations indicates that the population may be differentiated due to genetic drift. *Nm* > 1 between populations indicates that the level of gene flow between populations is higher and the genetic differentiation between populations is smaller. When *Nm* > 4, the gene exchange between populations is more sufficient and the genetic differentiation is more smaller (Wright 1951, 1978). In this study, we found that the *Nm* values of the four populations were less than 1, indicating that geographic isolation has completely hindered the gene exchange of the four populations. Compared with Qinghai Lake *G. przewalskii* and Yellow River *G. eckloni*, the genetic distance between Heihe River *G. chilianensis* and Shule River *G. chilianensis* was further and the differentiation degree was more significant.

The Maximum Likelihood (ML) phylogenetic trees showed that the populations of *G. chilianensis* (HH) clustered on one branch, *G. chilianensis* (SL) clustered on one branch, *G. eckloni* (YL) and *G. przewalskii* (QH) clustered on the other branch, and the bootstrap values of which were 83–99%. *G. chilianensis* had further genetic distance with *G. eckloni* and *G. przewalskii*. *G. chilianensis* should be recognized as an independent species, which is widely accepted (Wu et al. 1964; Li and Chang 1974; Zhang et al. 2013; Zhang et al. 2015), but Zhao (1991) suggested that *G. chilianensis* as a subspecies of *G. eckloni*. The results of this study provide a theoretical reference for their views. The results of this study also suggest the genetic distance among *G. przewalskii* and *G. eckloni* was closer (0.0100). The established Maximum Likelihood (ML) phylogenetic tree also showed that *G. przewalskii* and *G. eckloni* clustered into one branch and haplotype crossed each other.

Eighty-two haplotypes we observed were clustered into two clades and cross distribution each other (Figure 2). Clustering results in network haplotypes analysis were completely consistent with its clustering results in phylogenetic analysis, clustering results in haplotype network analysis can clearly indicate the relationship between four populations. Two populations of *G. chilianensis* (HH&SL) have developed genetic differentiation, but there was some cross distribution between haplotypes, indicating that the degree of genetic differentiation is not high. Other haplogroup contain *G. eckloni* (YL) and *G. przewalskii* (QH), and some haplotype cross distribution each other. In conclusion, *G. przewalskii* and *G. eckloni* might be subspecies of each other, however, further studies are required. The result of this study shows that the *G. chilianensis* (HH&SL) had lower genetic diversity than *G. przewalskii* (QH) and *G. eckloni* (YL). We suggest strengthen the protection of genetic resources of *G. chilianensis*. According to different groups, effective protection measures should be set up to actively protect the ecological environment in the basin and improve its habitat environment.

## Data Availability

The data that support the findings of this study are available in figshare at https://doi.org/10.6084/m9.figshare.13122809.v1
